# Multiple preputial stones: A case report and literature review

**DOI:** 10.1016/j.ijscr.2020.04.041

**Published:** 2020-05-11

**Authors:** Muhammad Asykar Palinrungi, Khoirul Kholis, Syakri Syahrir, Syarif, Muhammad Faruk

**Affiliations:** a Division of Urology, Department of Surgery, Faculty of Medicine, Hasanuddin University, Makassar, Indonesia; b Department of Surgery, Faculty of Medicine, Hasanuddin University, Makassar, Indonesia

**Keywords:** Preputial stone, Smegma, Infection, Phimosis, Circumcision, Case report

## Abstract

**Introduction:**

Preputial stones are a very rare form of urinary tract stone, and only small number cases have been reported in the literature, and tend to occur in uncircumcised males with poor genital hygiene, low socioeconomic status, and phimosis.

**Presentation of case:**

Here, we report a case in a male who presented with more than 100 preputial stones. The stones were evident on clinical examination by palpation on the preputial. The patient was treated by dorsal slit circumcision.

**Discussion:**

Preputial stones can occur at any age but are far more common in adult males. All cases of preputial stone are associated with severe phimosis in uncircumcised males. The symptoms and signs are due to phimosis, like in these patients, which causes urinary stasis beneath the foreskin. The stone is often palpable on examination of the prepuce, and a plain radiograph can confirm this. Neglected preputial stones can cause serious morbidities. Treatment involves the removal of stone and elimination of the predisposing cause.

**Conclusions:**

This case reminds us of the necessity of circumcision for adult uncircumcised males.

## Highlights

• Preputial stones are a very rare form of urinary tract stone.
• Almost all the cases happen in a male in uncircumcised males, poor genital hygiene, low socioeconomic status, and phimosis.
• The symptoms and signs are due to phimosis.
• Neglected preputial stones can cause serious morbidities, such as hydronephrosis and renal failure.

## 1 Introduction

Preputial stones are a very rare form of urinary tract stone, and few cases have been reported in the literature [1], occurring especially in uncircumcised males [2] with poor genital hygiene, and low socioeconomic status [3]. The first report of a preputial stone in an adult was by Robert Clarke in 1794 [4]. Preputial stone is primarily regarded as a result of severe phimosis; other causes are smegma solidification and accumulation of urine flow on the preputial area [1]. Here we report a case of an adult male with multiple preputial stones, in line with the updated consensus-based surgical case report (SCARE) guidelines [5].

## 2 Case presentation

**Fig. 1:**
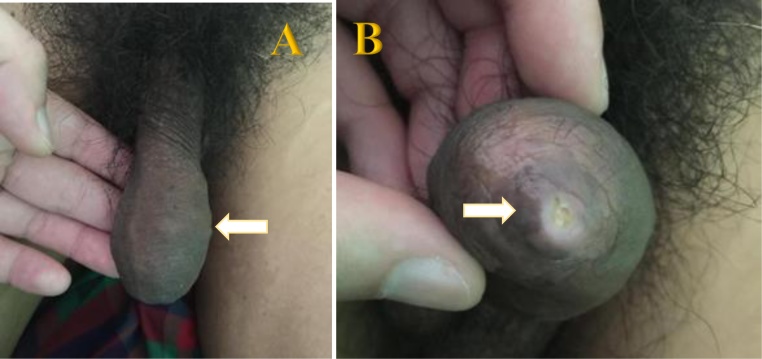
A. Gross appearance of the penis. B. Phimosis on examination (arrow).

**Fig. 2:**
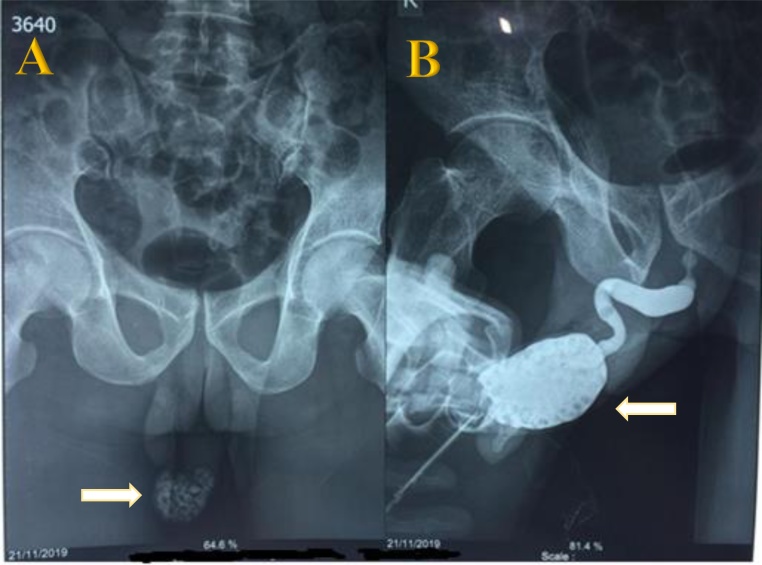
A. Plain film showing the multiple preputial stones (arrow). B. Urethrography x-ray showing multiple radio-opaque shadows in the tip of the penis (arrow), a normal caliber urethra with no evidence of stricture.

**Fig. 3:**
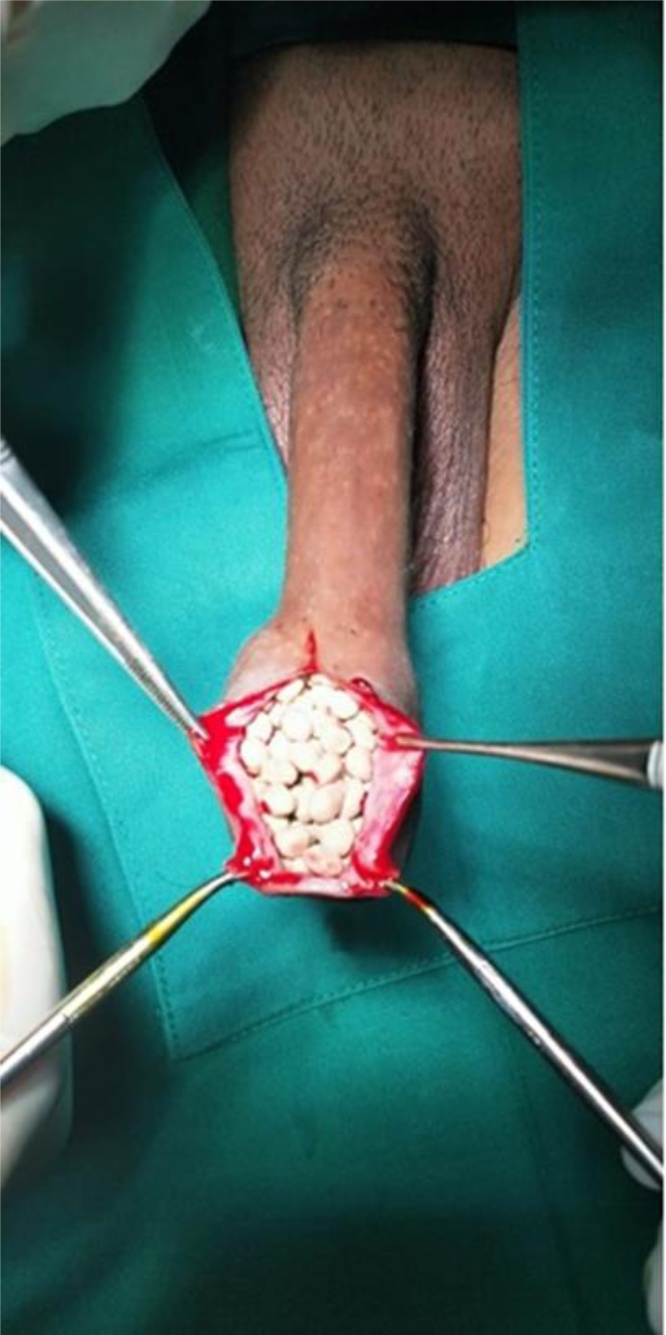
After the dorsal incision, multiple white stones were apparent in the preputial cavity.

**Fig. 4:**
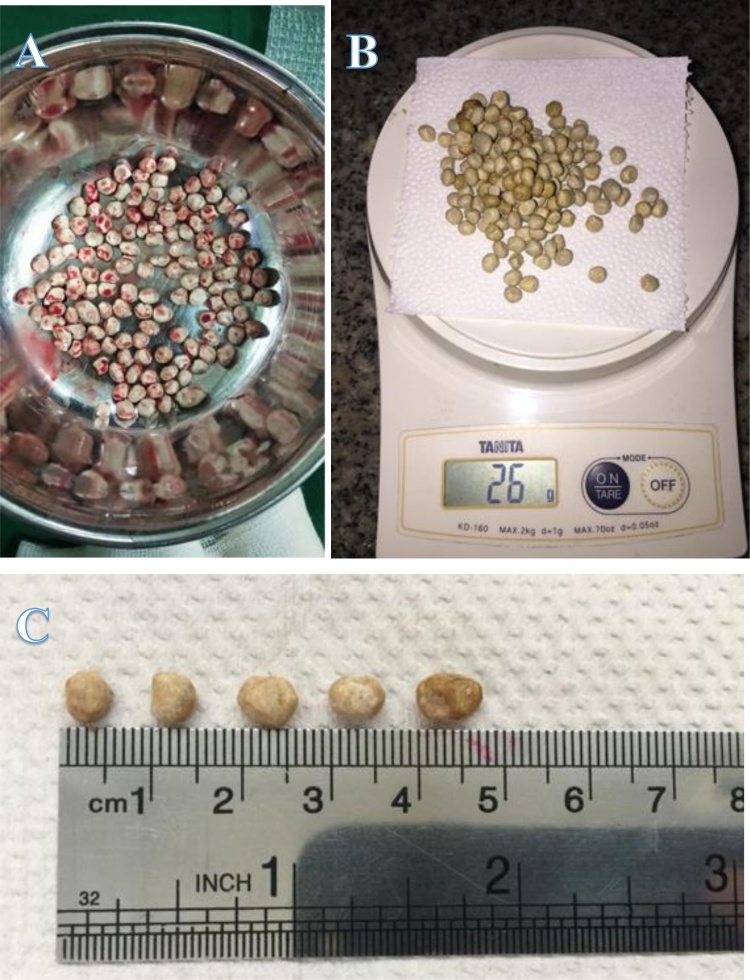
A. Multiple extracted stones. B. The total weight of the stones was 26 g. C. Multiple stones, ranging from 4 to 8 mm.

A 50-year-old man came to an outpatient clinic with the chief complaint a mass at the tip of the penis and progressive difficulty voiding for the past year, with a history of passing a stone on 48 occasions. Vital signs were within normal limits. On physical examination, the prepuce appeared to be phimosis and was palpable, with a thick preputial skin and stone inside the preputial cavity (Fig. 1). On upper tracts ultrasound, serum creatinine level and other biochemical parameters were within normal limits. Urinalysis revealed 10–14 leukocytes/high-power field (HPF) on microscopic examination. A plain film and urethrography x-ray showed multiple radio-opaque shadows in the tip of the penis, with a normal caliber of the urethra, and no evidence of stricture (Fig. 2). Dorsal slit circumcision and preputial stone extraction were done (Fig. 3), recovering 134 stones of up to 4 × 8 mm (Fig. 4). The stone analysis revealed 44% carbonate apatite phosphate, 38% ammonium urate, 10% amorphous calcium phosphate carbonate, and 8% matrix (unknown matter).

## 3 Discussion

Preputial stones can occur at any age but are far more common in adult males [6]. In Indonesia, childhood circumcision is a traditional practice; which likely accounts for this being the first reported case of preputial stones in Indonesia. All cases of preputial stone are associated with severe phimosis in uncircumcised males [1]. Stones usually occur singularly or as a few; only five cases have reported the presence of more than 100 stones (Table 1).

**Table 1:** Comparison of our case with other literature.

No.	Authors/year of publication	Age (year)	Chief complaint	Obstructive uropathy	Causative factor	Characteristics of stone	Composition of stone	Surgery
1.	Present case	50	Mass at the tip of the penis	No	Phimosis	Multiple stones, ranging from 4 to 8 mm; the total weight of the stones was 26 g	Carbonate apatite phosphate, ammonium urate, amorphous calcium phosphate carbonate	Dorsal slit circumcision
2.	Tze Huat Chong et al. [7]	27	difficulty in passing urine and leaked urination	Yes	Phimosis	A single stone, measured 50 × 50 mm	NA	
3.	Gajanan S. Bhat [6]	65	Mass at the tip of the penis	Yes	Phimosis	Twenty-five stone ranging from 4 to 15 mm	Calcium phosphate	Dorsal slit circumcision
4.	Kekre et al. [2]	11	continuous urine leakage with history meningomyelocele and placement of VP shunt	NA	Phimosis	Multiple stones; total weight, 9.96 g	Uric acid, urates, phosphates, xanthine, calcium, magnesium, oxalate, and ammonia.	Circumcision
5.	Spataru RI et al. [9]	5	incontinence for urine in a history of myelomeningocele operation	No	Phimosis	A single stone, 3–2 cm	Calcium oxalate	Circumcision
6.	Tuğlu D et al. [1]	12	Urinary tract infection with preputial skin fistula in a history of myelomeningocele operation	No	Phimosis	Multiple stones ranging from 1 to 2 cm	NA	Dorsal slit circumcision
7.	Yuasa et al. [10]	92	Acute urinary retention with obstructive uropathy	Yes	Phimosis	Multiple sized stones; total weight, 100 g	NA	Dorsal slit circumcision
8.	Nagata D et al. [11]	32	Painless macroscopic haematuria	NA	Phimosis	Multiple stones	Magnesium ammonium phosphate, calcium phosphate, and calcium carbonate	Circumcised
9.	Mohapatra TP et al. [12]	65	Progressive difficulty in voiding and foul-smelling penile discharge with cancer of the penis	No	Phimosis	Multiple, faceted stone	Calcium ammonium magnesium Phosphate, Magnesium calcium urate	Partial penectomy
10.	Ellis DJ et al. [3]	4	whitish penile discharge and progressive difficulty in voiding.	No	Post epispadias repair, foreign body induced calculus	A single stone, 14 × 18 mm	Ammonium acid urate, magnesium ammonium phosphate hexahydrate	Extracted under general anesthesia
11.	Kim SO et al. [6]	NA	NA	No	Phimosis; Associated with bladder calculi and TCC of bladder	NA	NA	NA
12.	Sharma SK [6]	NA	NA	NA	Phimosis	NA	NA	NA
13.	Sharma SK et al. [6]	NA	NA	NA	NA	NA	NA	NA
14.	Shahi UN et al. [1]	2 cases: (1) 55 (2) 60	(1) Acute urinary retention; (2) Dribbling of urine	NA	Phimosis	(1) Two stones; diameters, 2.5 and 0.7 cm (2) Five stones; diameter, 1–2 cm	Calcium, magnesium, phosphate, carbonate, and urate	Circumcision
15	Wilford EC [6]	NA	NA	NA	Phimosis	NA	Sodium and calcium phosphate	NA

NA: Data not available.

The symptoms and signs are due to phimosis, which causes urinary stasis beneath the foreskin [3]. In some cases, the urinary obstruction can be severe, causing obstructive uropathy [6]. Preputial stones might be associated with complications, such as dysuria, stranguria, hematuria, and preputial ballooning during voiding, rarely with urinary retention [2], obstructive uropathy, foul-smelling discharge from prepuce [6], and preputial skin fistula [1].

Metabolic evaluation can provide clues about the cause of stone formation, especially in a situation where the stone is found in the other parts of the urinary tract, such as the kidney, ureter, and bladder (KUB) [6]. The stones are often palpable on examination of the prepuce; however, a plain radiograph can confirm the existence [7]. Ultrasound or KUB, or both, are essential to rule out any proximal stones, as the treatment will be either minimally invasive (e.g., shock wave lithotripsy) or involve endoscopic or open surgery [7].

Wilford characterized preputial stones according to their pathogenesis [3]: 1) inspissated smegma with lime salts, 2) struvite composition secondary to an infection, and 3) stone formed in the proximal urinary tract, which is trapped during migration. Winsbury-White characterized preputial stones by their composition [3]: 1) inspissated smegma, 2) smegma and urinary salts, 3) and urinary salts alone. In our case, the stones were mostly composed of carbonate apatite phosphate and ammonium urate, thus indicating a combination of a nidus of smegma acting as a condensation nucleus for the precipitation of urinary salts and urinary tract infection. Smegma is an accumulation of cellular debris in the preputial fold and has a dual role in preputial stone formation [8]. In addition to functioning as a nidus, smegma can be a direct irritant, inducing inflammation, adhesions, and preputial stenosis, and leading to obstruction with stasis [3].

Treatment involves the removal of stones and elimination of the predisposing cause [7]. As in this case, the patient underwent a dorsal slit circumcision procedure to remove the stone. Neglected preputial stones might cause serious morbidities, such as hydronephrosis and renal failure secondary to obstructive uropathy [1,7] and preputial skin fistula [1] (Table 1).

## 4 Conclusion

Preputial stones occur primarily in adults with phimosis and poor hygiene. Factors contributing to urinary tract stone formation, including obstruction, stasis, infection, and nidus deposition, are implicated in the genesis of preputial stone. Our findings support the necessity of circumcision for adult uncircumcised males.

## Declaration of Competing Interest

Nothing to declare.

## Funding

No funding or sponsorship.

## Ethical approval

The study is exempt from ethical approval in our institution.

## Consent

Written informed consent was obtained from the patient for publication of this case report and accompanying images.

## Author contribution

**Muhammad Asykar Palinrungi and Muhammad Faruk:** study concept, surgical therapy for this patient. **Syakri Syahrir:** Data collection, Writing - Original draft preparation. **Khoirul Kholis: s**enior author and the manuscript reviewer. **Syarif:** reviewed the manuscript. **Muhammad Faruk:** Editing, Writing. All authors read and approved the final manuscript.

## Registration of research studies

Not applicable – single case report.

## Guarantor

Muhammad Asykar Palinrungi.

## Provenance and peer review

Editorially reviewed, not externally peer-reviewed.
